# Prevalence of Obesity and Overweight Among Children in Aseer Region, Saudi Arabia

**DOI:** 10.3390/jcm15010076

**Published:** 2025-12-22

**Authors:** Youssef A. Alqahtani, Ayed A. Shati, Ashwag A. Asiri, Samy A. Dawood, Yazan A. Almaker, Abdulmajeed F. AlShahrani, Asma A. Nasser, Seham M. Alqahtani

**Affiliations:** 1 Department of Child Health, College of Medicine, King Khalid University, Abha 62521, Saudi Arabiaasalasiri@kku.edu.sa (A.A.A.);; 2 Pediatrics Department, Khamis Mushayt Maternity and Children’s Hospital, Khamis Mushait 62454, Saudi Arabia; 3 College of Medicine, King Khalid University, Abha 62521, Saudi Arabia

**Keywords:** childhood obesity, overweight, Aseer Region, Saudi Arabia, Body Mass Index (BMI), fast food, physical activity

## Abstract

**Background:** Childhood overweight and obesity represent growing public health challenges globally, driven by complex interactions between demographic, behavioral, and familial factors. This study assessed the prevalence and determinants of overweight and obesity among school-aged children and adolescents attending urban schools in the Aseer Region of Saudi Arabia (Abha and Khamis Mushait). **Methods:** A cross-sectional study was conducted among 413 urban school students aged 6–18 years. Anthropometric measurements were obtained using standardized procedures, and lifestyle behaviors were assessed through a structured questionnaire. Data were analyzed using descriptive statistics, Chi-square tests, and multivariate binary logistic regression. Variables with *p* < 0.05 in univariate analysis, alongside conceptually relevant predictors, were included in the final model. Adjusted odds ratios (AORs) with 95% confidence intervals (CIs) were reported. **Results:** The combined prevalence of overweight and obesity was high, particularly among older age groups and secondary-school students. Significant associations were found between BMI category and age, school level, parental education, and family history of obesity. Lifestyle behaviors including fast-food consumption, low fruit and vegetable intake, prolonged screen time, and short sleep duration showed strong relationships with overweight/obesity. In the adjusted model, key predictors included frequent fast-food intake (AOR = 2.74), low fruit/vegetable intake (AOR = 2.20), physical inactivity (AOR = 1.70), high screen time (AOR = 2.40), short sleep duration (AOR = 1.55), and positive family history of obesity (AOR = 3.10). **Conclusions:** Childhood overweight and obesity in the Aseer Region are influenced by both modifiable lifestyle behaviors and familial predisposition. Targeted interventions promoting healthy dietary habits, adequate physical activity, reduced screen time, and sufficient sleep are essential. Preventive strategies should prioritize adolescents and children with a family history of obesity to effectively reduce obesity risk.

## 1. Introduction

Childhood overweight and obesity have increased rapidly over recent decades and now represent a major public-health challenge worldwide [1,2,3]. In 2022, over 390 million children and adolescents aged 5–19 years were estimated to be overweight, including a large and growing number living with obesity, and the prevalence of overweight among this age group increased from about 8% in 1990 to approximately 20% in 2022 [4]. Global studies estimate that the prevalence of obesity in children and adolescents is substantial and rising, with projections indicating a marked increase in the number of young people affected in the coming years [5,6,7]. For example, pooled analyses estimate an obesity prevalence in children and adolescents of approximately 8.5% (95% CI 8.2–8.8) and project that the number of children and adolescents living with obesity will increase substantially by 2025 and 2030 [8].

The World Health Organization recommends using BMI-for-age references to classify weight status among school-aged children and adolescents and to monitor trends across populations; these standards underpin international surveillance and comparability of prevalence estimates [9]. Beyond population counts, global reports indicate that the burden of overweight and obesity among children has major implications for future population health because excess adiposity in childhood is strongly associated with metabolic risk, cardiovascular disease, and persistence of obesity into adulthood [4].

In the Kingdom of Saudi Arabia, childhood overweight and obesity have risen to levels that merit urgent attention. Systematic reviews of national studies report wide but consistently high prevalence estimates, with overweight and obesity proportions varying substantially across regions and age groups; review data indicate that overweight in children ranges roughly from 5% to 29%, while obesity estimates range from approximately 3.8% to 49.7% depending on the population and classification used [10]. Regional analyses also show that older children and adolescents and male students commonly register higher prevalence and that lifestyle behaviors, particularly frequent fast-food consumption, physical inactivity, and prolonged screen time, are repeatedly implicated as modifiable drivers [11,12].

Despite these insights, gaps remain in the literature. Many prior studies have examined single behaviors or limited age groups, and relatively few have simultaneously assessed a broad set of demographic, dietary, activity, sleep, screen-time, and familial variables in urban school populations of the Aseer Region. Understanding the combined influence of these factors in an urban Saudi setting is essential for designing effective, locally tailored prevention programs.

Therefore, this study aimed to estimate the prevalence of overweight and obesity among school-aged children attending urban schools in Abha and Khamis Mushait and to identify demographic, lifestyle, and familial factors associated with overweight/obesity.

## 2. Materials and Methods

### 2.1. Study Design and Setting

A school-based cross-sectional study was conducted in March 2025 in the urban centers of Abha and Khamis Mushait, Aseer Region, Saudi Arabia. According to the latest available data, the two cities together include approximately 436 schools (215 in Abha and 221 in Khamis Mushait) [13]. All selected schools were in urban areas; rural schools were not included.

### 2.2. Study Population

The target population consisted of children and adolescents aged 6–18 years enrolled in primary, intermediate, and secondary schools within the selected cities. The inclusion criteria were: (1) students aged between 6 and 18 years, (2) current enrollment in one of the selected schools, and (3) provision of informed parental consent and child assent. Children with physical disabilities or known endocrine or genetic disorders that could affect growth and BMI were excluded from the study.

### 2.3. Sampling Procedure

A multistage stratified random sampling technique was employed to ensure representative coverage.

- Stage 1: Two major cities (Abha and Khamis Mushait) were selected.
- Stage 2: From each city, both public and private schools were randomly selected to achieve equitable representation.
- Stage 3: Within each selected school, students were stratified by educational level (primary, intermediate, secondary), and then randomly chosen from school enrolment lists using simple random sampling.

This approach ensured adequate representation of both genders, multiple age groups, and socioeconomic categories.

### 2.4. Sample Size Determination

The minimum sample size was calculated using the formula:

$$n=Z^{2}*\frac{p(1-p)}{d^{2}}$$

where *Z* = 1.96 (for 95% confidence level), *p* = 0.50 (assumed prevalence), and *d* = 0.05 (margin of error). The estimated minimum sample size was 384. To account for potential non-response or incomplete data, 30% was added, giving a final target sample of 500 students. Ultimately, 413 valid responses were analyzed.

### 2.5. Data Collection Procedure

Data were collected through two main components:

1. Anthropometric measurements
2. Structured questionnaires

#### 2.5.1. Anthropometric Assessment

Anthropometric measurements (height and weight) were performed following standard procedures recommended by the WHO and expert anthropometry guidelines. Standing height was measured using a portable stadiometer (Seca 213, Seca GmbH, Hamburg, Germany), with participants barefoot, heels together, and head positioned in the Frankfurt plane. Body weight was measured using a calibrated digital weighing scale (Omron HN-289, Omron Healthcare Co., Ltd., Kyoto, Japan). Each measurement was taken in duplicate and the average was recorded, in line with recommended best practices for field studies [14]. Body Mass Index (BMI) was calculated as:

$$BMI=\frac{Weight\;(kg)}{Height\;{\left(m\right)}^{2}}$$

BMI (kg/m^2^) was then calculated and converted to BMI-for-age percentiles using WHO 2007 growth reference data, as implemented in WHO AnthroPlus software (version 1.0.4; World Health Organization, Geneva, Switzerland); the globally recommended tool for assessing nutritional status in children and adolescents aged 5–19 years [15].

According to WHO cutoffs, children with BMI-for-age <5th percentile were classified as underweight, those between 5th and 84th percentile as normal weight, those between 85th and 94th percentile as overweight, and those ≥95th percentile as obese [16].

#### 2.5.2. Questionnaire

A structured, interviewer-administered questionnaire was used to collect information on sociodemographic and lifestyle variables. The tool was adapted from validated instruments in previous studies [12,17] and translated into Arabic. It comprised four sections:

1. Demographic information: Age, gender, school level, parental education, and employment.
2. Health and family background: Presence of chronic diseases and family history of obesity.
3. Dietary habits: Frequency of fast-food and sugary-drink consumption, fruit and vegetable intake, and breakfast patterns.
4. Lifestyle behaviors: Physical activity frequency, screen time, and average sleep duration.

To ensure validity, the questionnaire was reviewed by a panel of public health and pediatric experts. A pilot test was conducted with 20 participants, and minor modifications were made to improve clarity. Data from the pilot were excluded from the final analysis.

### 2.6. Data Management and Statistical Analysis

All collected data were coded and entered into Microsoft Excel, cleaned, and subsequently analyzed using IBM SPSS Statistics version 26.0 (IBM Corp., Armonk, NY, USA). Descriptive statistics were used to summarize participants’ characteristics and BMI distribution. Categorical variables were presented as frequencies and percentages, while continuous variables were expressed as means and standard deviations (SD). Associations between BMI categories and demographic or lifestyle variables were examined using Chi-square (χ^2^) tests, a significance threshold of *p* < 0.05 was applied for all bivariate comparisons.

Variables were selected for multivariate logistic regression based on conceptual relevance as well as statistical significance. All variables with *p* < 0.05 in univariate χ^2^ tests were included. In addition, variables considered epidemiologically important predictors of childhood obesity from previous literature (breakfast habit, physical activity, parental education, and family history of obesity) were also included regardless of their univariate *p*-values, to avoid residual confounding. This approach is consistent with established recommendations for multivariable model building.

### 2.7. Ethical Considerations

Ethical approval for this study was obtained from the Research Ethics Committee at King Khalid University (Approval No. ECM#2025-702; HAPO-06-B-001). The study was conducted in accordance with the ethical principles outlined in the Declaration of Helsinki. Permission was obtained from the Aseer Region Education Authority and participating schools prior to data collection. Written informed consent was obtained from the parents or guardians of all participating children, and assent was obtained from the students themselves. All collected data were treated with strict confidentiality and used solely for research purposes.

## 3. Results

### 3.1. Prevalence of BMI Categories

Table 1 shows several important demographic patterns associated with BMI categories among the 413 participating children. Although gender differences did not reach statistical significance (*p* = 0.117), males exhibited a higher proportion of obesity (22.1%) compared with females (13.4%), suggesting a possible trend toward greater risk in boys. In addition to the high burden of overweight and obesity, a substantial proportion of participants were classified as underweight. Overall, 27.4% of males and 30.5% of females were categorized as underweight, respectively. Age demonstrated a very strong association with BMI (*p* < 0.001), with obesity and overweight increasing markedly in older age groups. Children aged 6–12 years had the lowest obesity prevalence (11.2%), whereas adolescents aged 16–18 years had the highest combined overweight and obesity burden (59.0%), indicating a progressive rise in excess weight with advancing age.

A similar pattern was observed across academic levels (*p* < 0.001), where secondary-school students exhibited substantially higher rates of overweight (32.9%) and obesity (24.8%) than primary-school students. Parental education was also significantly associated with BMI for both fathers (*p* = 0.014) and mothers (*p* = 0.016). Children whose parents particularly mothers had higher education levels tended to have higher obesity rates, possibly reflecting socioeconomic and lifestyle differences in urban households.

Family history of obesity showed a strong and statistically significant association with BMI (*p* < 0.001). Among children who were obese, 65.3% reported a positive family history, compared with only 36.6% among normal-weight children. This highlights a substantial familial or genetic influence. Chronic disease status did not reach statistical significance (*p* = 0.201), although obese children had a slightly higher proportion of chronic conditions.

### 3.2. Association Between Lifestyle Factors and Overweight/Obesity

Table 2 demonstrates the relationship between lifestyle behaviors and BMI status. Fast-food consumption was strongly associated with BMI category (*p* < 0.001). Children consuming fast food more than four times per week had markedly higher rates of overweight (25.0%) and obesity (21.3%) compared with those who consumed it less frequently, emphasizing the role of calorie-dense diets in weight gain. Fruit and vegetable intake also displayed a clear inverse relationship with obesity (*p* < 0.001). Low intake (<2 times/week) was common among overweight (67.0%) and obese children (58.7%), while high intake (>4 times/week) was more frequent among normal-weight children.

Breakfast habit showed no statistically significant association (*p* = 0.168), but the pattern indicates that irregular breakfast consumption corresponds to higher obesity rates (61.3%), supporting existing evidence that breakfast skipping may contribute to unhealthy weight. Physical activity also demonstrated a nonsignificant trend (*p* = 0.108), with children engaging in <60 min of daily activity showing higher rates of obesity (65.3%) compared to their more active counterparts.

Screen time exhibited a significant association with BMI (*p* = 0.025). Excessive screen exposure (>3 h/day) was reported by 80.7% of overweight and 70.7% of obese children. Similarly, sleep duration displayed one of the strongest associations (*p* < 0.001). Short sleep (<8 h/day) was most prevalent among overweight (51.1%) and obese children (56.0%), whereas longer sleep (8–10 h/day) was more common among normal-weight participants.

### 3.3. Logistic Regression Analysis of Risk Factors

Table 3 presents the effects of demographic and lifestyle factors on the odds of overweight/obesity. Children consuming fast food more than four times per week had significantly increased odds of overweight/obesity (AOR = 2.74), even after adjusting for other variables. Moderate fast-food consumption (2–4 times/week) also increased risk (AOR = 1.88). Similarly, inadequate fruit and vegetable intake was a strong predictor; consuming fruits/vegetables <2 times per week doubled the odds of excess weight (AOR = 2.20).

Irregular breakfast habits were associated with a 45% increased risk (AOR = 1.45, *p* = 0.024), while low physical activity (<60 min/day) increased the odds by 70% (AOR = 1.70, *p* = 0.007). High screen time was another strong predictor, with children exceeding 3 h/day having more than twice the odds of overweight/obesity (AOR = 2.40). Short sleep duration (<8 h/day) increased risk by 55% (AOR = 1.55).

Family history of obesity emerged as the strongest predictor overall, tripling the odds of overweight/obesity (AOR = 3.10, *p* < 0.001). Parental education also showed modest associations, with children of fathers (AOR = 1.35) or mothers (AOR = 1.40) holding bachelor’s degrees showing higher odds compared with postgraduate-educated parents.

## 4. Discussion

In this study, we observed a high burden of overweight and obesity among school-aged children in urban centers of the Aseer Region, with prevalences of 21.3% (overweight) and 18.2% (obesity). These figures are within the upper range reported by previous studies in Saudi Arabia. A recent systematic review covering 21 studies across the country found that overweight among children ranged from 5% to 29%, and obesity ranged from 3.8% to 49.7% depending on region, age group, and classification criteria [10]. This underscores that childhood obesity remains a serious public-health challenge in the Kingdom.

Our finding that male students had a higher prevalence of obesity than females aligns with some study indicating that obesity tends to be more frequent among boys [11,18,19,20]. The observed increase in overweight/obesity with age and academic level also corresponds to earlier evidence suggesting that older children and adolescents are more susceptible to excess weight gain, likely due to cumulative exposure to obesogenic lifestyle factors [8,21,22,23].

One of the most important findings from the multivariate model is that several lifestyle behaviors including fast-food consumption, fruit and vegetable intake, breakfast habits, screen time, sleep duration, and physical activity were independently associated with overweight/obesity. Frequent fast-food consumption, particularly ≥4 times per week, showed the strongest dietary association, nearly tripling the odds of overweight/obesity. This aligns with studies demonstrating that energy-dense fast foods contribute significantly to caloric surplus and weight gain among children [24,25,26,27].

Low fruit and vegetable intake (<2 times/week) doubled the odds of overweight/obesity, a finding consistent with global evidence indicating that poor dietary quality is a key driver of childhood obesity [28,29]. Irregular breakfast consumption also remained a significant predictor. This supports multiple studies showing that skipping breakfast disrupts appetite regulation and leads to overeating later in the day.

Screen time and sleep duration emerged as strong behavioral predictors. Children exceeding 3 h/day of screen exposure had more than twice the odds of overweight/obesity, and those sleeping <8 h/day had substantially higher risk; findings consistent with literature linking sedentary behaviors and insufficient sleep to dysregulated metabolism, increased snacking, and reduced physical activity [30,31].

Family history of obesity showed the strongest association in the multivariate model, tripling the odds of excess weight. This finding highlights the combined effects of genetic susceptibility and shared family behaviors, echoing patterns previously documented in Saudi children [32]. Parental education remained a modest predictor, suggesting that higher socioeconomic status in urban households may coexist with increased access to calorie-dense foods and sedentary entertainment options, potentially offsetting the advantages of higher health literacy.

An additional finding of this study is the relatively high prevalence of underweight, the use of WHO BMI-for-age standards may classify constitutionally lean but otherwise healthy children as underweight, particularly in populations with variation in growth patterns and body composition. Also, the cross-sectional design and reliance on self-reported dietary behaviors may introduce measurement limitations.

An important strength of this study is the concurrent consideration of multiple modifiable behaviors; diet, physical activity, sleep, screen time along with familial factors. This multifactorial approach offers a more comprehensive view of childhood obesity determinants than studies focusing solely on single variables.

Nevertheless, certain limitations must be acknowledged. First, the study sample comprised urban children only (schools in Abha and Khamis Mushait); hence, results cannot be generalized to rural populations in the region, where dietary patterns, physical activity levels, and socio-economic conditions may differ substantially. Second, although we collected data on key lifestyle behaviors, we did not assess consumption of sugary beverages or sweet and salty snacks, which are common contributors to obesity; the absence of these variables may lead to underestimation of dietary risk factors. Third, chronic disease status was self-reported or extracted from school health records, which may be incomplete; the influence of chronic illnesses on BMI and lifestyle behaviors should be interpreted cautiously. Fourth, the cross-sectional design precludes causal inference; although associations are robust, temporal direction and causality remain unconfirmed. Finally, although we used WHO BMI-for-age percentiles (via AnthroPlus), BMI does not account for body composition (e.g., fat mass vs. lean mass), and thus may misclassify some children. Also, BMI does not distinguish between fat mass and lean mass and may misclassify muscular children, especially adolescents participating in sports. More accurate adiposity measures such as body fat percentage or waist-to-height ratio would better differentiate between healthy weight and excess fat. Finally, Lifestyle behaviors were self-reported without a defined retrospective timeframe, which may introduce recall bias and reduce accuracy of exposure measurement.

Given these findings, several public health implications emerge. There is urgent need for school-based and community-level health promotion programs in urban areas of the Aseer Region, focusing on reducing fast-food consumption, encouraging regular breakfast, increasing fruit/vegetable intake, limiting screen time, improving physical activity, and promoting healthy sleep hygiene. In addition, parental and family-centered interventions are key, considering the strong influence of family history and possibly familial behaviors. Policymakers might consider regulations or awareness campaigns to reduce exposure to high-calorie fast foods accessible to children. Moreover, future research should expand to rural areas and include more comprehensive dietary assessments (e.g., sugary drinks, snacks), and ideally longitudinal designs to track changes over time.

## 5. Conclusions

This study demonstrates that overweight and obesity among school-aged children and adolescents are influenced by a combination of demographic, familial, and lifestyle factors. Older age, positive family history of obesity, frequent fast-food consumption, low intake of fruits and vegetables, irregular breakfast habits, physical inactivity, prolonged screen time, and short sleep duration were all identified as significant predictors of excess weight. These findings reinforce the multifactorial nature of childhood obesity and highlight the importance of addressing unhealthy behavioral patterns alongside familial predispositions.

Given that many of the identified risk factors are modifiable, the results underscore the need for comprehensive, school- and family-based interventions that promote balanced nutrition, regular physical activity, healthy sleep routines, and reduced sedentary behavior. Public health strategies should prioritize early prevention, particularly among high-risk groups such as adolescents and children with a family history of obesity. Future research incorporating longitudinal designs and more accurate measures of adiposity will help clarify causal pathways and further inform targeted interventions aimed at reducing the growing burden of childhood obesity.

## Figures and Tables

**Table 1:** BMI Category Distribution by Demographic Characteristics.

Variable	Total *n* (%)	Underweight *n* (%)	Normal Weight *n* (%)	Overweight *n* (%)	Obese *n* (%)	χ^2^ (*p*-Value)
**Gender**						5.882 (0.117)
Male	226 (54.7)	62 (27.4)	71 (31.4)	43 (19.0)	50 (22.1)	
Female	187 (45.3)	57 (30.5)	60 (32.1)	45 (24.1)	25 (13.4)	
**Age group**						101.532 (<0.001)
6–12 years	188 (45.5)	96 (51.1)	46 (24.5)	25 (13.3)	21 (11.2)	
13–15 years	74 (17.9)	12 (16.2)	34 (45.9)	9 (12.2)	19 (25.7)	
16–18 years	151 (36.6)	11 (7.3)	51 (33.8)	54 (35.8)	35 (23.2)	
**Academic level**						89.713 (<0.001)
Primary	188 (45.5)	95 (50.5)	45 (23.9)	26 (13.8)	22 (11.7)	
Intermediate	76 (18.4)	14 (18.4)	33 (43.4)	13 (17.1)	16 (21.1)	
Secondary	149 (36.1)	10 (6.7)	53 (35.6)	49 (32.9)	37 (24.8)	
**Father’s Education**						20.685 (0.014)
Illiterate	12 (2.9)	0 (0.0)	9 (6.9)	1 (1.1)	2 (2.7)	
Before university	97 (23.5)	25 (21.0)	38 (29.0)	22 (25.0)	12 (16.0)	
Bachelor’s	277 (67.1)	83 (69.7)	80 (61.1)	59 (67.0)	55 (73.3)	
Postgraduate	27 (6.5)	11 (9.2)	4 (3.1)	6 (6.8)	6 (8.0)	
**Mother’s Education**						20.255 (0.016)
Illiterate	40 (9.7)	5 (4.2)	14 (10.7)	7 (8.0)	14 (18.7)	
Before university	172 (41.6)	49 (41.2)	60 (45.8)	40 (45.5)	23 (30.7)	
Bachelor’s	210 (50.8)	60 (50.4)	57 (43.5)	45 (45.5)	48 (48.0)	
Postgraduate	8 (1.9)	5 (4.2)	0 (0.0)	1 (1.1)	2 (2.7)	
**Mother’s Employment**						1.512 (0.679)
Employed	145 (35.1)	41 (34.5)	46 (35.1)	35 (39.8)	23 (30.7)	
Unemployed	268 (64.9)	78 (65.5)	85 (64.9)	53 (60.2)	52 (69.3)	
**Family History of Obesity**						
Yes	213 (51.6)	69 (58.0)	48 (36.6)	47 (53.4)	49 (65.3)	19.457 (<0.001)
No	200 (48.4)	50 (42.0)	83 (63.4)	41 (46.6)	26 (34.7)	
**Chronic Disease**						
Yes	122 (29.5)	41 (34.5)	34 (26.0)	20 (22.7)	27 (36.0)	15.781 (0.201)
No	291 (70.5)	78 (65.5)	97 (74.0)	68 (77.3)	48 (64.0)	

**Table 2:** Lifestyle Factors Associated with BMI Categories.

Variable	Category	Total *n* (%)	Underweight *n* (%)	Normal *n* (%)	Overweight *n* (%)	Obese *n* (%)	χ^2^ (*p*-Value)
**Fast food intake**	<2 times/week	142 (34.4)	49 (41.2)	56 (42.7)	16 (18.2)	21 (28.0)	23.321 (<0.001 **)
	2–4 times/week	200 (48.4)	49 (41.2)	63 (48.1)	50 (56.8)	38 (50.7)	
	>4 times/week	71 (17.2)	21 (17.6)	12 (9.2)	22 (25.0)	16 (21.3)	
**Fruit and Vegetable Intake**	<2 times/week	252 (61.0)	86 (72.3)	63 (48.1)	59 (67.0)	44 (58.7)	26.348 (<0.001 **)
	2–4 times/week	142 (34.4)	29 (24.4)	64 (48.9)	26 (29.5)	23 (30.7)	
	>4 times/week	19 (4.6)	4 (3.4)	4 (3.1)	3 (3.4)	8 (10.7)	
**Breakfast Habit**	Regular	247 (59.8)	69 (58.0)	85 (64.9)	64 (72.7)	29 (38.7)	5.049 (0.168)
	Irregular	166 (40.2)	50 (42.0)	46 (35.1)	24 (27.3)	46 (61.3)	
**Physical Activity**	≥60 min/day	196 (47.5)	59 (49.6)	67 (51.1)	44 (50.0)	26 (34.7)	6.078 (0.108)
	<60 min/day	217 (52.5)	60 (50.4)	64 (48.9)	44 (50.0)	49 (65.3)	
**Screen time**	≤3 h/day	129 (31.2)	46 (38.7)	44 (33.6)	17 (19.3)	22 (29.3)	9.333 (0.025 *)
	>3 h/day	284 (68.8)	73 (61.3)	87 (66.4)	71 (80.7)	53 (70.7)	
**Sleep Duration**	<8 h/day	166 (40.2)	29 (24.4)	50 (38.2)	45 (51.1)	42 (56.0)	31.316 (<0.001 **)
	8–10 h/day	232 (56.2)	88 (73.9)	76 (58.0)	40 (45.5)	28 (37.3)	
	>10 h/day	15 (3.6)	2 (1.7)	5 (3.8)	3 (3.4)	5 (6.7)	

* Significant at *p* < 0.05; ** Significant at *p* < 0.01.

**Table 3:** Multivariate Logistic Regression Model of Predictors of Overweight/Obesity.

Variable	Category	AOR (95% CI)	*p*-Value
**Fast food intake**	<2 times/week	Ref	
	2–4 times/week	1.88 (1.32–2.63)	0.001 **
	>4 times/week	2.74 (1.69–4.44)	<0.001 **
**Fruit and Vegetable Intake**	>4 times/week	Ref	
	2–4 times/week	1.90 (1.21–3.32)	0.007 **
	<2 times/week	2.20 (1.42–3.84)	<0.001 **
**Breakfast Habit**	Regular	Ref	
	Irregular	1.45 (1.03–2.00)	0.024 *
**Physical Activity**	≥60 min/day	Ref	
	<60 min/day	1.70 (1.10–2.50)	0.007 **
**Screen time**	≤3 h/day	Ref	
	>3 h/day	2.40 (1.95–2.96)	<0.001 **
**Sleep Duration**	≥8 h/day	Ref	
	<8 h/day	1.55 (1.08–2.30)	0.026 *
**Family history of obesity**	No	Ref	
	Yes	3.10 (2.50–4.18)	<0.001 **
**Father’s education**	Postgraduate	Ref	
	Bachelor’s	1.35 (1.01–1.80)	0.043 *
	Before university	1.21 (0.78–1.78)	0.316
	Illiterate	1.44 (0.41–4.41)	0.411
**Mother’s education**	Postgraduate	Ref	
	Bachelor’s	1.40 (1.04–1.87)	0.021 *
	Before university	1.22 (0.82–1.69)	0.309
	Illiterate	1.55 (0.93–2.33)	0.081

* Significant at *p* < 0.05; ** Significant at *p* < 0.01.

## Data Availability

The data supporting the findings of this study are available from the corresponding author upon reasonable request. Due to ethical and privacy restrictions imposed by the Institutional Review Board, individual-level data cannot be made publicly accessible.

## Abbreviations

The following abbreviations are used in this manuscript:

BMI	Body Mass Index
WHO	World Health Organization
IRB	Institutional Review Board
SPSS	Statistical Package for the Social Sciences
